# Biogeography in *Cellana* (Patellogastropoda, Nacellidae) with Special Emphasis on the Relationships of Southern Hemisphere Oceanic Island Species

**DOI:** 10.1371/journal.pone.0170103

**Published:** 2017-01-18

**Authors:** Claudio A. González-Wevar, Tomoyuki Nakano, Alvaro Palma, Elie Poulin

**Affiliations:** 1 GAIA-Antártica, Universidad de Magallanes, Punta Arenas, Chile; 2 Instituto de Ecología y Biodiversidad (IEB), Departamento de Ciencias Ecológicas, Facultad de Ciencias, Universidad de Chile, Ñuñoa, Santiago, Chile; 3 Seto Marine Biological Laboratory, Field Science Education and Research Centre, Kyoto University, Nishimuro, Wakayama, Japan; 4 Universidad Gabriela Mistral, Facultad de Ingeniería y Negocios, Providencia, Santiago, Chile; Australian Museum, AUSTRALIA

## Abstract

Oceanic islands lacking connections to other land are extremely isolated from sources of potential colonists and have acquired their biota mainly through dispersal from geographically distant areas. Hence, isolated island biota constitutes interesting models to infer biogeographical mechanisms of dispersal, colonization, differentiation, and speciation. Limpets of the genus *Cellana* (Nacellidae: Patellogastropoda) show limited dispersal capacity but are broadly distributed across the Indo-Pacific including many endemic species in isolated oceanic islands. Here, we examined main distributional patterns and geographic boundaries among *Cellana* lineages with special emphasis in the relationships of Southern Hemisphere oceanic islands species. Phylogenetic reconstructions based on mtDNA (COI) recognized three main clades in *Cellana* including taxa from different provinces of the Indo-Pacific. Clear genetic discontinuities characterize the biogeography of *Cellana* and several lineages are associated to particular areas of the Indo-Pacific supporting the low dispersal capacity of the genus across recognized biogeographical barriers in the region. However, evolutionary relationships within *Cellana* suggest that long-distance dispersal processes have been common in the history of the genus and probably associated to the origin of the species in Hawaii and Juan Fernández Archipelago. Therefore, the presence of *Cellana* species in geographically distant Southern Hemisphere oceanic islands, such as the Juan Fernández Archipelago, suggests that long-distance dispersal mediated by rafting may have played an important role in the biogeography of the genus.

## Introduction

The distribution of near-shore benthic invertebrates is the result of complex interactions between historical and contemporary processes as well as biological traits including physiological adaptations, reproduction modes, and especially their dispersal capacities [1,2]. The movement and dispersal of organisms is a fundamental component of nearly all ecological and evolutionary processes [3]. In general, oceanic dispersal correlates to larval lifespan and oceanographic conditions, which strongly conditions the geographical range, and the genetic structure of populations [1,4]. The transport of larvae in ocean currents is a major mechanism of dispersal for many planktotrophic marine organisms, whose larvae may spend months, or even years, drifting in the plankton [5]. However, many widely distributed marine species are direct developers or possess short-lived larvae [4,6,7]. The failure to predict the extent of a species distribution from its larval lifespan suggests that either undescribed biological factors or historical constraints are of paramount importance [8]. In this context, long-distance dispersal mediated by rafting on drifting material plays a major role in the colonization of geographically isolated areas [9,10]. Even when the relevance of long-distance dispersal is fairly understood, the rarity and unpredictability of these events has precluded the development of testable hypothesis [10–12].

**Fig 1:**
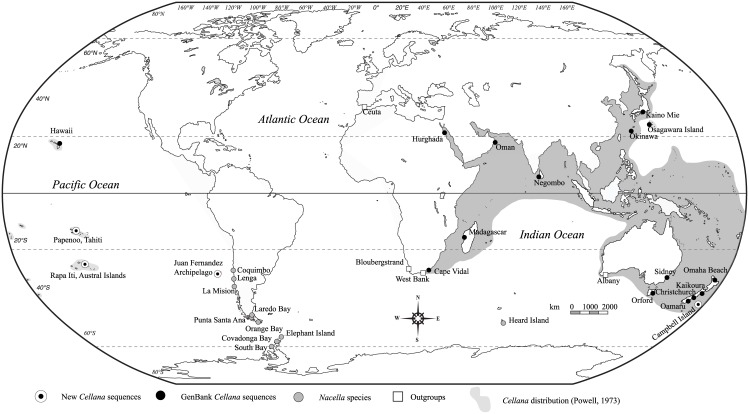
Current distribution of *Cellana* along the Indo-Pacific showing sampling localities of *Cellana*, *Nacella* and Outgroups.

True limpets of the order Patellogastropoda are key near-shore marine benthic elements of inter- and sub-tidal rocky ecosystems [13]. The Nacellidae includes two genera, *Cellana* and *Nacella*, with markedly disjoint distributions in the Indo-Pacific and the Southern Ocean, respectively. *Cellana* includes more than 35 species and several subspecies distributed in tropical and warm-temperate waters across Indo-Pacific [14]. In general, nacellid limpets exhibit short larval lifespan (< 20 days) compared to other gastropods. In spite of these short larval lifespan, several nacellids are endemic to isolated islands [14]. Across the Southern Hemisphere, *Cellana* expands its distribution to several Pacific Islands including those from Micro-, Mela-, and Polynesia. The eastward distribution of the genus includes the Juan Fernández Archipelago, located ~ 700 km off the coast of Chile [14] and more than 6000 kms from its closest relatives currenltly found in the Marquesas and Austral islands (Fig 1).

The main objective of this study is to determine the relationships of Southern Hemisphere oceanic island *Cellana* species including those from Tahiti, French Polynesia, Juan Fernández Archipelago and Campbell Island, subantarctic New Zealand. Through phylogenetic reconstructions we aim to derive a clear picture concerning the evolutionary relationships in *Cellana* and the taxonomic status of different populations. Divergence time estimates will permit us to determine how historic and contemporary processes have shaped the biogeography of *Cellana*. Addressing these issues in this dominant benthic group will provide new information about the biogeography of *Cellana* in isolated oceanic islands of the Southern Hemisphere.

## Methods

### Ethics statement

This work was conducted using true limpets of the genus *Cellana* as model study. The species *Cellana ardosidea*, endemic of the Juan Fernández Archipelago is not protected and is not included in the Chilean fishery statistic. Permission to undertake field works and to collect specimens was issued by the Chilean Fishery Service Director (María Angela Barbieri), under the technical memorandum (011/2010). The Instituto de Ecología y Biodiversidad (IEB/15-2015) and Chilean Fishery Service (SERNAPESCA 011/2010) Ethics Committees approved sampling protocols and experiments. For this, we complied with local legislation and the Convention on Biological Diversity.

### Sampling, DNA preparation, PCR amplification and alignment

Individuals of *C*. *ardosidea* were collected from two localities at Robinson Crusoe Island, Juan Fernández Archipelago, El Palillo (33°38′S; 78°49′W) and El Inglés (33°37′S; 78°49′W; Fig 1). Specimens of *C*. *strigilis strigilis* specimens were collected in Perseverance Harbour, Campbell Island (52°32′S; 169°8′ E; Fig 1). *Cellana dira* individuals were collected in Rapa Iti, Austral Islands, French Polynesia (27°35′S; 144°20′W), and *C*. *taitensis* specimens were sampled in Papenoo, Tahiti (17°35'S; 149°26'W) and Orofara, Tahiti (17°30'S; 149°28'W; Table 1). Identification of all species with the exception of *C*. *dira* was done following Powell [14]. The species *C*. *dira* was previously included in the genus *Patella* but external features such as gill morphology and inner shell coloration suggest that this taxon belongs to *Cellana* (T. N., pers. obs.).

**Table 1:** Patellogastropod species, specimens, accession numbers, and sampling localities included in the study. Specimen identifications were based on Powell [14], Valdovinos and Rüth [70], Nakano and Ozawa [17], and González-Wevar et al. [18]. New COI sequences obtained in this study are marked in bold. Other Accession Numbers and their respective sources are indicated.

Species	Localities	N°	DNA ID	COI
*Nacellidae*				
*Nacella concinna*, Strebel 1908	South Bay, Ant. Pen.	1	NCON-05	GU901239.1
	Elephant Island, Ant. Pen.	1	NCON-44	GU901240.1
	Covadonga Bay, Ant. Pen.	1	NCON-63	GU901242.1
*Nacella clypeater*, Lesson, 1831	La Misión, Chile	1	NCLY-07	GU901248.1
	Lenga, Chile	1	NCLY-23	GU901250.1
	Coquimbo, Chile	1	CLY-45	GU901252.1
*Nacella deaurata*, Gmelin, 1791	Laredo Bay, Patagonia	3	NDEA-09	GU901233.1
			NDEA-21	GU901234.1
			NDEA-34	GU901235.1
*Nacella magellanica*, Gmelin, 1791	Laredo Bay, Patagonia	2	NMAG-04	GU901229.1
	Orange Bay, Patagonia	1	NMAG-10	GU901230.1
			NMAG-28	GU901232.1
*Nacella mytilina*, Helbling, 1779	Puerto del Hambre, Patagonia	3	NMYT-05	GU901243.1
			NMYT-24	GU901244.1
			NMYT-35	GU901245.1
*Nacella flammea*, Gmelin, 1779	Puerto del Hambre, Patagonia	3	NFLA-01	GU901253.1
			NFLA-04	GU901254.1
			NFLA-06	GU901255.1
*Nacella kerguelenensis*, E.A. Smith, 1877	Heard Island	3	NKER-01	GU901224.1
			NKER-02	GU901225.1
			NKER-03	GU901226.1
*Nacella macquariensis*	Heard Island	3	NMAC-01	GU901219.1
			NMAC-02	GU901220.1
			NMAC-03	GU901221.1
*Cellana eucosmia*, Pilsbry, 1891	Hurghada, Egypt	1	NUGB-L396	AB238543
*Cellana flava*, Hutton, 1873	Kaikoura, New Zealand	1	NUGB-L576	AB238545
*Cellana radians*, Gmelin, 1791	Omaha Beach, New Zealand	1	NUGB-L580	AB238551
*Cellana radiata enneagona* Reeve, 1854	Madagascar	1	NUGB-L490	AB238553
*Cellana radiata orientalis* Pilsbry, 1891	Okinawa, Japan	1	NUGB-L27	AB238554
*Cellana radiata capensis* Gmelin, 1891	Cape Vidal, South Africa	1	NUGB-L395	AB238552.1
*Cellana radiata radiata*, Born, 1778	Negombo, Sri Lanka	1	NUGB-L515	AB238559.1
*Cellana solida*, Brainville, 1825	Orford, Tasmania, Australia	1	NUGB-L401	AB238561
*Cellana testudinaria*, Linnaeus, 1758	Okinawa, Japan	1	NUGB-L42	AB238563
*Cellana toreuma*, Reeve, 1854	Oga Akita, Japan	1	NUGB-L3	AB238564
*Cellana tramoserica*, Holten, 1802	Botany Bay, Sydney, Australia	1	NUGB-L647	AB238566
*Cellana stellifera*, Gmelin, 1791	Christchurch, New Zealand	1	NUGB-L740	AB433648-1
*Cellana denticulada*, Martyn, 1784	Kaikoura, New Zealand	1	NUGB-L575	AB238544.1
*Cellana karachiensis* Winckworth, 1930	Oman	1	UF:292785B	AB433634.1
*Cellana nigrolineata*, Reeve, 1839	Kaiko Mie, Japan	1	NUGB-L51	AB548213.1
*Cellana mazatlandica* Sowerby, 1839	Ogasawara Islands, Japan	1	NUGB-L717	AB433635-1
*Cellana talcosa*, Gould, 1846	Hawaii	4	Mol LAU M177	EF621038.1
			Mol LAU M178	EF621039.1
			Mol LAU M180	EF621041.1
			Mol LAU M181	EF621042.1
*Cellana sandwicensis*, Pease, 1861	Hawaii	3	Mol LAU M167	EF621299.1
			Mol LAU M2013	EF621300.1
			Mol LAU M2014	EF621301.1
*Cellana exarata*, Reeve, 1854	Hawaii	3	NWH NR NR120	EF621189.1
			NWH NR NR123	EF621191.1
			NWH NR NR124	EF621192.1
*Cellana strigilis redimiculum*, Reeve, 1854	Oamaru, New Zealand	1	NUGB-L741	AB433649.1
*Cellana ardosidea*, Hombron & Jaquinot, 1841	Juan Fernández Archipelago	5	CEAR-01	**KY353124**
			CEAR-04	**KY353125**
			CEAR-05	**KY353126**
			CEAR-11	**KY353127**
			CEAR-13	**KY353128**
*Cellana strigilis strigilis* Hombron & Jaquinot	Campbell Island, New Zealand	5	CSTR-01	**KY353129**
			CSTR-02	**KY353130**
			CSTR-03	**KY353131**
			CSTR-04	**KY353132**
			CSTR-05	**KY353133**
*Cellana dira*	Rapa Iti, Austral Islands	4	NUGB-L1164	**KY353120**
			NUGB-L1165	**KY353121**
			NUGB-L1166	**KY353122**
			NUGB-L1167	**KY353123**
*Cellana taitensis*, Röding, 1798	Papenoo, Tahiti	2	NUGB-L1050	**KY353116**
	Orofara, Tahiti	2	NUGB-L1051	**KY353117**
			NUGB-L1052	**KY353118**
			NUGB-L1053	**KY353119**
*Patellidae*				
*Cymbula oculus*, Born, 1778	West Bank, South Africa	1	NHM	AB238572
*Helcion concolor*, Krauss, 1848	West Bank, South Africa	1	NHM	AB238574
*Helcion dunkeri*, Krauss, 1848	Cape Town, South Africa	1	NHM	AB238575
*Patella caerulea*, Linnaeus, 1758	Ceuta, Spain	1	NUGB-L653	AB238577
*Patella ferruginea*, Gmelin, 1758	Ceuta, Spain	1	NUGB-L655	AB238578
*Patella rustica*, Linnaeus, 1758	Ceuta, Spain	1	NUGB-L651	AB238579
*Scutellastra laticostata*, Blainville, 1825	Albany, Australia	1	NUGB-L659	AB238584

Animals were fixed in ethanol (95%), and total DNA was extracted from the mantle following the salting-out protocol described by Aljanabi and Martinez [15]. A partial fragment of the mitochondrial gene Cytochrome Oxidase c Subunit I (COI) was amplified using universal primers [16]. PCR mixtures and thermal cycling parameters were done following Nakano and Ozawa [17] and González-Wevar et al. [18]. PCR amplicons were purified using QIAquick Gel Extraction Kit (QIAGEN) and both strands were sequenced using an Automatic Sequencer 3730xl at Macrogen Inc. (South Korea). New COI sequences of *Cellana* species were deposited in GenBank under the following Accession Numbers: KY353116 –KY353133.

### Phylogenetic reconstructions

We included in the analyses four to five individuals of each of the newly analyzed *Cellana* species. New COI sequences were combined with previously published COI sequences of *Cellana* available in GenBank (Table 1). COI sequences of *Nacella* species and four patellogastropod genera (*Helcion*, *Scutellastra*, *Cymbula*, and *Patella*) were used as sister- and out-group, respectively. New *Cellana* sequences were edited using Proseq 2.91 [19] and the whole COI sequence data set was aligned with Clustal W [20]. Sequences were translated to amino acids to check for the presence of pseudogenes, insertion/deletions (indels), and/or the presence of sequencing errors with MEGA 6.0 [21]. Reconstructions were done using three different methods: Maximum Parsimony (MP), Maximum Likelihood (ML), and Bayesian Inference (BI). MP and ML analyses were performed in PAUP* version 4.0b [22] following González-Wevar et al. [18]. Nucleotide substitution models for ML and BI were selected using the Akaike Information Criterion (AIC) and the Bayesian Information Criterion (BIC), with jmodeltest 2.0 [23], respectively. ML and BI analyses were performed using PHYML [24] and MrBayes 3.1.2 [25], respectively. Inferred tree nodes supports for MP and ML analyses were assessed through a non-parametric bootstrap (BS) analysis with the full heuristic search option and 1,000 pseudo-replicates [26]. Bayesian analyses were used to obtain posterior probabilities for the nodes in the phylogenetic trees. Posterior probability values of sampled trees were obtained using the Metropolis coupled Markov-Chain Monte-Carlo algorithm (MCMC) implemented in MrBayes. Four chains were run twice in parallel for 100 x 10^6^ generations and trees were sampled every 1,000 generations. Stationarity of the analyses was inferred when the average standard deviation of split frequencies was less than 0.01 [25]. The first 10% of the trees were discarded (burn-in) and Bayesian posterior probabilities (BPP) were estimated as the percentage of trees that showed a particular node. Nodes in the phylogenetic tree were considered highly supported with BPP values ≥ 0.95% and BS ≥ 75%. Nodes with support values of 0.80–0.95% (BPP) and 60–75% (BS) were considered moderately supported. Posterior probability density of the combined tree and log files was summarized as a maximum clade credibility tree using TreeAnnotator v.1.6.1 (http://beast.bio.ed.ac.uk/TreeAnnotator) and then visualized with FigTree v.1.4 (http://tree.bio.ed.ac.uk/software/figtree).

### Divergence time estimates

Divergence time estimates were made using a relaxed molecular clock analyses with an uncorrelated lognormal (ucln) model of molecular evolutionary rate heterogeneity and the GTR + I + G substitution model in BEAST v.1.6.2 [27]. For this, we used a specific mutation rate (0.85%– 1.15%) previously estimated for nacellid limpets [28]. An age prior with a normal distribution was applied to the most recent common ancestor (tmrca) of Nacellidae (mean, 38; SD, 3.8), an Upper Eocene fossil of *Cellana ampla* [29]. We also include two age priors within *Nacella*, a tmrca of *N*. *concinna* (mean, 4.5; SD, 0.45) based on the oldest fossil record for this species at Cockburn Island [30] and the tmrca of *N*. *clypeater* (mean, 4, SD, 0.4) based on *N*. *clypeater*-like fossil from southern Peru [31]. A birth-death speciation prior was used for branching rates in the phylogeny. Four chains were run two times for 100 x 10^6^ generations, and trees were sampled every 1,000 generations. Majority rule consensus phylograms, as well as posterior probabilities for nodes were estimated. Convergence of model parameter values and estimated node heights to their optimal posterior distribution were estimated by plotting the marginal posterior probabilities versus the generation state in Tracer v.1.5 (http://beast.bio.ed.ac.uk/Tracer). Effective sample size values were estimated for each parameter to ensure adequate mixing of the Markov-Chain Monte-Carlo (MCMC ESSs > 1000).

## Results

### Data analyses and phylogenetic reconstructions

Sequence alignment for COI included 647 nucleotide positions, with 271 variable and parsimony informative sites. As expected for a coding region, no indels or stop codons were detected and no significant levels of saturation were observed in the complete COI data set. Out of a possible 215 amino acids in the entire COI data set of *Cellana* we identified eleven amino acid substitutions, all of them occurred at the third position of the codon.

**Fig 2:**
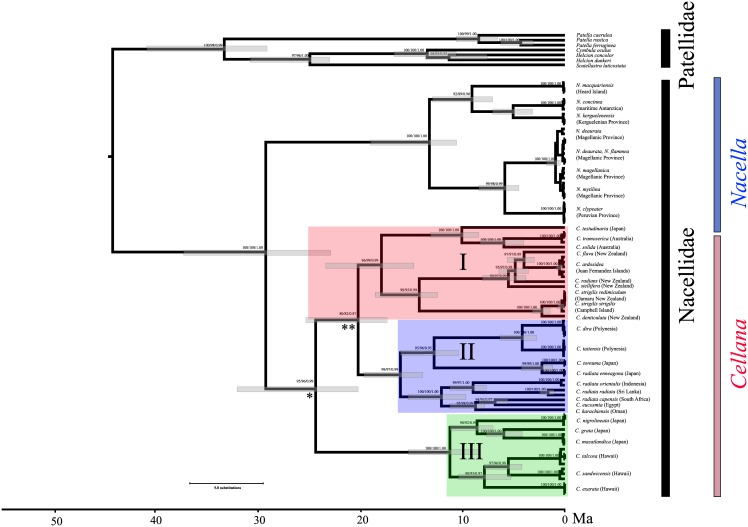
Bayesian maximum credibility tree (100 x 10^6^ generations trees sampled every 1000 generations) in the evolution of the Patellogastropoda. Support values and posterior probabilities are marked in each node MP/ML/BI. Scale at the x-axis represents estimated age in million years and grey bars represent 95% highest posterior density intervals. * indicates the separation of the major clades (A, B, and C) within *Cellana* and ** indicates the separation between clades A and B.

**Fig 3:**
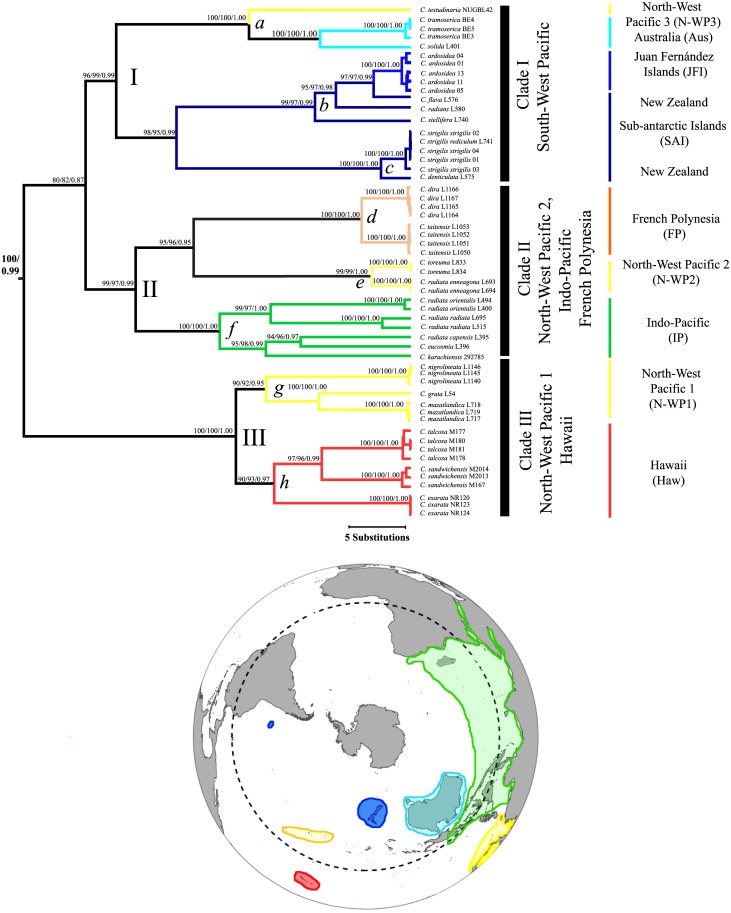
Bayesian maximum credibility tree (100 x 10^6^ generations trees sampled every 1000 generations) showing the recognized clades in the evolution of *Cellana*. Each clade was named according to the current distribution of their representatives.

COI sequences clearly discriminated major taxonomic groupings of patellogastropods with high bootstrap (BS) and Bayesian posterior probabilities (BPP) (Fig 2). At the same time, all the reconstruction methods confirmed the monophyly of both *Nacella* and *Cellana*, and their sister relationships (BS = 100%; BPP = 1.0; Fig 2). Within *Cellana*, we recorded three main clades (I, II, and III; Figs 2 and 3). Clade I includes species from Australia, New Zealand, *C*. *ardosidea* from Juan Fernández archipelago, and *C*. *testudinaria* from Japan. Clade II comprises species from the Indo-Pacific, the Northwestern Pacific and French Polynesia. Finally, Clade III contains species from the Northwestern Pacific and Hawaii. Each one of these major clades can also be divided into subclades (*a*–*h*; Fig 3) containing species from particular areas. For instance, subclade *a* includes Australian *C*. *tramoserica* and *C*. *solida* (Aus) and *C*. *testudinaria* from the Northwestern Pacific (N-WP3; Fig 3). Subclade *b* includes species from New Zealand (*C*. *flava*, *C*. *radians*, and *C*. *stellifera*) and *C*. *ardosidea* from Juan Fernández Island (JFI), while subclade *c* comprises *C*. *denticulata* from Kaikoura New Zealand, and nominal subspecies *C*. *strigilis redimiculum* and *C*. *strigilis strigilis* from New Zealand Subantarctic Island (SAI; Fig 3). Within main clade II, subclade *d* includes *C*. *dira* (Austral Island) and *C*. *taitensis* (Tahiti) from French Polynesia (FP) while subclade *e* comprises *C*. *toreuma* and *C*. *radiata enneagona* from Japan (N-WP2). Subclade *f* includes species from the Indo-Pacific (IP) such as *C*. *radiata orientalis* (Indonesia), *C*. *radiata radiata* (Sri Lanka), *C*. *capensis* (South Africa), *C*. *eucosmia* (Egypt), and *C*. *karachiensis* (Oman). Subclade *g* includes *C*. *nigrolineata* and *C*. *grata* from Japan (N-WP1), *C*. *mazatlandica* (Osagawara Island), and subclade *h* comprises *C*. *exarata*, *C*. *talcosa* and *C*. *sandwicensis* from Hawaii (Haw; Fig 3).

New phylogenetic reconstructions based on mtDNA sequences are in basic agreement with previous studies in *Cellana* and support the idea that the oldest representatives of the genus correspond to the New Zealand species [17,32,33]. The position of *C*. *dira* within *Cellana* was confirmed in all reconstructions (T. N., pers. comm.; Figs 2 and 3). Following this, the species *Cellana dira* is now listed in World Register of Marine Species: WoRMS (http://www.marinespecies.org).

### Divergence time estimates

The separation of *Nacella* and *Cellana* took place close to the Eocene-Oligocene boundary around 30 Ma (37–24 Ma) (Fig 2). The most recent common ancestor (tmrca) of *Cellana* existed ~ 24 Ma (32–20 Ma) and is older than the 14 Ma (18–10 Ma) estimated for *Nacella*’s trmca. The separation of the three main clades is dated close to the Oligocene/Miocene boundary (∼ 24 Ma). After this, the main lineages of *Cellana* followed different evolutionary trajectories and diversified in different areas of the Indo-Pacific since the early Miocene. In the Southern Hemisphere, the diversification of *Cellana* in New Zealand North Island (*C*. *radians*, *C*. *stellifera* and *C*. *solida*) is placed ∼ 6 Ma (8–3 Ma). Then, the separation of Juan Fernández *C*. *ardosidea* from its closest New Zealand relative *C*. *flava* took place ~4.5 Ma (6.5–4 Ma). Finally, the separation between *C*. *denticulata* from New Zealand South Island and the two nominal subspecies of the *C*. *strigilis* complex is placed around 2.5 Ma (3.0–1.5 Ma). The separation between Polynesian *C*. *dira* and *C*. *taitensis* and the two species from the Northwestern Pacific (N-WP2) *C*. *toreuma* and *C*. *radiata enneagona* is placed ~12 Ma (16–10 Ma). Then, the separation between *C*. *dira* and *C*. *taitensis* took place ~4 Ma (6–3 Ma) (Fig 2).

## Discussion

### Historical biogeography of *Cellana*

Divergence time analyses suggest that the most recent common ancestor of the Nacellidae occurred around 30 Ma, close to the Eocene-Oligocene boundary, a period of marked worldwide tectonic, oceanographic and climatic changes, particularly in the Southern Ocean [34,35]. Since the Eocene, a global cooling trend is observed commonly explained by the combined effect of gradual northward movement of continents, the closure of the Tethys Sea, and the isolation of Antarctica and the initiation of the Antarctic Circumpolar Current [36,37]. Considering these settings, we propose that the cladogenetic process separating tropical/temperate *Cellana* from Antarctic/subantarctic *Nacella* coincides with the shift to glacial conditions in the Southern Ocean [38,39]. Consequently, the diversification of both genera could be related to important adaptations to the thermal realms where they currently occur.

Divergence time estimates in *Cellana* suggest that the origin and diversification of the genus across the Eastern Pacific Barrier (EPB) initiated near the Oligocene-Miocene boundary, 10 Ma earlier than previously estimated [18]. The EPB has been in place during the last 65 Ma [40] but transoceanic gene flow between populations at its two edges was potentially possible until the closure of the Tethyan Sea (11–17 Ma) or probably as recently as the definitive closure of the Panama Isthmus around 3.1 Ma [41]. Based on phylogenetic relationships and the distribution of lineages in *Cellana*, the Northwestern Pacific region seems to represent a key area in the biogeography of the genus because representatives of this region are present in the all three main clades.

### Oceanic island colonization in *Cellana*

**Fig 4:**
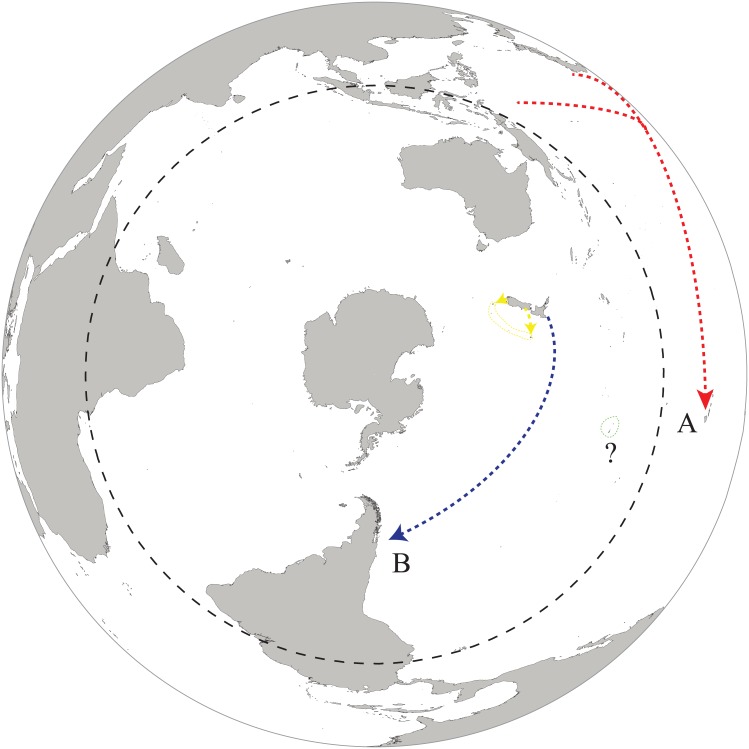
Long-distance dispersal events in the biogeography of *Cellana*. A) red line, colonization of Hawaii (Haw) during the Miocene from a North-Western Pacific source (N-WP1); B) blue line, colonization of Juan Fernández Archipelago (JFI) during the Mio-Pliocene from a New Zealand source (NZ), yellow arrow head, colonization of New Zealand sub-Antarctic Islands (SAI) during the Plio-Pleistocene from a New Zealand South Island source (NZSI). Whether the colonization of French Polynesia represents a long-distance dispersal event or a stepping-stone mediated process is still uncertain (?).

Evolutionary relationships within *Cellana* and divergence time analyses suggest the existence of long-distance dispersal events in the biogeography of the genus at different temporal and spatial scales. As previously demonstrated and discussed by Bird et al. [42], the colonization of the Hawaiian Islands represents an example of long-distance dispersal from the Northwestern Pacific Region (subclade N-WP1, Figs 3 and 4B). The monophyly of the Hawaiian species strongly supports the existence of a single colonization event [42]. In this context, a report of *Cellana* individuals living on the trunks of floating palm trees [43] support the hypothesis of rafting as a feasible mechanism for long-distance dispersal. Similarly, based on evolutionary relationships and divergence time estimations the colonization of Juan Fernández archipelago during the Mio-Pliocene (5.5 to 3 Ma) from the geographically distant New Zealand could also be associated to a long-distance dispersal event (Fig 4C). The presence of a single lineage (*C*. *ardosidea*) in Juan Fernández archipelago supports the hypothesis of a single dispersal event. Divergence time estimates are in basic agreement with the geologic ages of these islands [44]. Several examples corroborate the affinity between Juan Fernández archipelago and Western Pacific biota including fishes [45,46], gastropods [47,48] and marine algae [49]. Although the Juan Fernández and Desventuradas archipelagos are only 600–700 km away from South America, they are isolated from this continent by the presence of the Humboldt Current System. Hence, these islands exhibit a high degree of endemicity for molluscan [50] and fish [46] faunas. As previously suggested for other Juan Fernandez intertidal marine organisms [49], it is unlikely that *Cellana* could have crossed the entire width of the Pacific Ocean solely by larval dispersal considering the short larval period recorded in different lineages of the genus. Either rafting or island hopping of now-vanished seamounts during low sea level or volcanic activity could be invoked as a plausible explanation for the close relationship between New Zealand and Juan Fernández Archipelago *Cellana* species.

Whether the colonization of French Polynesia represents an example of long-distance dispersal in *Cellana* or a stepping-stone mediated process is difficult to infer considering phylogenetic relationships within clade II. French Polynesian species (*C*. *taitensis* and *C*. *dira*) are related to broadly distributed *Cellana* species (N-WP2; Figs 3 and 4A) *C*. *radiata* and *C*. *toreuma* [14,17]. The *radiata* complex is currently distributed from East Africa to the Marquesas Islands in the Pacific while the *toreuma* complex extends from Japan to the Philippines [14]. Therefore, the presence of *C*. *radiata* subspecies and *C*. *toreuma* on nearby areas of Polynesia argues in favor a colonization of Tahiti and Austral Islands through a stepping-stone mode rather than by long-distance dispersal. Phylogenetic and phylogeographic relationships within the *Cellana strigilis* complex in New Zealand Subantarctic Islands *Cellana* species has been discussed by Reisser et al [51]. In this study, the absence of reciprocal monophyly and the presence of shared haplotypes between *C*. *strigilis redimiculum* (Oamaru, New Zealand South Island) [17] and *C*. *strigilis strigilis* from Campbell Island (this study) suggest the existence of contemporary connectivity by regular gene flow.

### Long-distance dispersal mechanisms in *Cellana*

Commonly, dispersal is inferred as the default explanation of biogeographical disjunction following rejection of a vicariance hypothesis, for example by molecular dating. Considering the volcanic origin of oceanic islands in the Pacific a vicariant hypothesis for the biogeography of *Cellana* is most unlikely. Following this, several hypotheses can account for the presence of *Cellana* species at isolated oceanic islands and their evolutionary affinities to geographically distant relatives [52]. They include: i) *planned or accidental anthropogenic dispersal*, ii) *oceanic epiplanktonic larval dispersal* and iii) *oceanic dispersal of adults or juveniles by rafting*. Altough a case of long distance artificial introduction of a patellogastropod species by a fouling or as larva in ballast waters has been reported [53], the divergence time estimates in *Cellana* analysed in this study suggest that an anthropogenic dispersal hypothesis is very unlikely. Patterns of genetic divergence recorded between oceanic island *Cellana* species and their closest relatives support a long period of separation rather than evidence of contemporary human-related connectivity. As typical patellogastropods, nacellid limpets exhibit short period of larval dispersal with a trochophore stage of 1–2 days and a veliger stage of 7–11 days [13]. Therefore, short larval lifespan recorded in *Cellana* species from different provinces [54,55] suggests that an oceanic epiplanktonic larval dispersal hypothesis is not likely to hold. In fact, larval-mediated dispersal in *Cellana* should not extend for more than a couple of hundred kms [56] and certainly not across wide stretches of ocean [1,6,8,41]. Otherwise, *Cellana* larvae would have to average more than 13 km/h to travel from New Zealand to Juan Fernández archipelago (considering a maximum larval duration of three weeks). Such estimations in terms of required current velocities are far higher than those estimated based on First Global GARP Experiment (FGGE) drifters for the ACC at the Drake Passage, the fastest current of the planet where velocities do not exceed 1.5 km/h [57]. In this context, island hopping could also constitute a plausible explanation for long distance dispersal in *Cellana*. Long-distance dispersal by island hopping has been reported in Northwestern Pacific *Cellana* species [33]. Therefore, the ancestral species could have extended its distributional range colonizing new appearing islands following sea level changes or volcanic activities. Following this, they could have become isolated and diversified by the loss of stepping stone ‘islands’ by further sea level changes or geologic activities. However, as proposed for other invertebrates [58], a hypothesis of island hopping between New Zealand and Juan Fernández is quite unlikely considering the distance between these areas, the degree of isolation of Juan Fernández, and by the absence of intervening islands.

In this context, macroalgae constitute the most important floating objects for grazers like *Cellana* in the Southern Hemisphere as they combine a relatively high food value with high floatation potential [59]. Particularly, brown algae represent suitable transportation vectors because they can travel along large distances mediated by oceanographic circulations in the Southern Ocean [60]. Absence of genetic structure in *Macrocystis pyrifera* and *Durvillaea antarctica* populations from New Zealand and South America supports the high dispersal capacity across the Southern Ocean [61–63]. Several groups of macroalgae are also currently found in New Zealand and Juan Fernández archipelago [50,64], supporting the possibility of long-distance dispersal of *Cellana* adults and/or juveniles via rafting to these islands. A rafting community study over *D*. *antarctica* recorded several macro-invertebrates including a living specimen of *C*. *strigilis strigilis* [59]. Molecular data estimated that these epibionts rafted for several weeks around 400 to 600 km (from Auckland/Snares Island to Otago in New Zealand). Considering the observations of these kelp-dwelling individuals of *Cellana* it is likely that macroalgae constitute suitable long-distance dispersal vectors to reach distant oceanic islands in the cold and temperate regions of the Southern Hemisphere. These observations in *Cellana* are in basic agreement with recent molecular studies providing strong evidence towards dispersal, but relatively little evidence for the biogeographic role of plate tectonic in the distribution of Southern Hemisphere marine benthic biota [9,52,59,60,65]. Accordingly, passive rafting should be considered as a possible contributory mechanism during investigations of the biogeographic processes involved in the current distribution of near-shore marine organisms.

Alternatively to this long-distance dispersal biogeographic hypothesis in the Southern Hemisphere, the Antarctic continent could also have played a role in the current distribution of oceanic island lineages. The climatic history of Antarctica during the last 50 Ma is quite complex and until the late Neogene (~ 23 Ma), this continent was much warmer and ice dominance was partial or ephemeral [34,66]. The development of ephemeral West Antarctic Ice Sheet occurred around 34 Ma coincident with the first East Antarctic ice sheets, but it was not a permanent feature until much later [67]. During the warmest intervals of the Pliocene (4.5–3.0 Ma), coinciding with the colonization of Juan Fernández archipelago, Earth’s average surface temperature was 2 to 3°C warmer than present [68]. During this warmth period of the Pliocene the West Antarctic Ice Sheet was reduced in extent therefore some ice-free littoral zones may have allowed *Cellana* to re-colonized this continent. From there, *Cellana* could have dispersed northward toward oceanic islands in the Pacific. Nevertheless, nacellid fossil records in Antarctica are sparse and include a late Eocene (~ 33 Ma) *Cellana*-like fossil (*C*. *feldmanni*) from la Meseta Formation, Seymour Island, Antarctic Peninsula [69] and Pliocene (~ 4.5 Ma) *Nacella concinna* individuals from Cockburn Island Formation, Antarctic Peninsula [30]. Nevertheless, the oldest *Cellana* fossil verified by shell microstructure includes *C*. *ampla* from the late Eocene (38 Ma) in Oregon, North America [29] while the oldest *Nacella* fossil lived during the late Oligocene at tropical latitudes in southern Peru [31]. Hence, the current nacellid Antarctic fossil record does not support the hypothesis of Antarctica as a staging post in the biogeography of Southern Hemisphere.
